# Applications of Focused Ultrasound in the Treatment of Genitourinary Cancers

**DOI:** 10.3390/cancers14061536

**Published:** 2022-03-17

**Authors:** John Panzone, Timothy Byler, Gennady Bratslavsky, Hanan Goldberg

**Affiliations:** Urology Department, SUNY Upstate Medical University, Syracuse, NY 13210, USA; panzonej@upstate.edu (J.P.); bylert@upstate.edu (T.B.); bratslag@upstate.edu (G.B.)

**Keywords:** ultrasound, cancer, treatment, review

## Abstract

**Simple Summary:**

Cancer is a prevalent disease globally, and conventional treatment options have been associated with substantial morbidity for patients. The unique acoustic properties and biological effects of focused ultrasound have been investigated for use as an alternative treatment option for various forms of cancer with lower associated morbidity than standard treatments. The objective of our review was to assess the current state and various applications of focused ultrasound for the treatment of genitourinary cancers, including prostate, kidney, bladder, penile, and testicular malignancies. Current research demonstrates that focused ultrasound-based focal therapy shows promise for the treatment of localized prostate and kidney cancer, and the effect of ultrasound on cell membranes may increase the efficacy of chemotherapeutics and radiotherapy. Focused ultrasound-based treatment modalities should continue to be investigated as an alternative or complementary treatment option for cancer patients.

**Abstract:**

Traditional cancer treatments have been associated with substantial morbidity for patients. Focused ultrasound offers a novel modality for the treatment of various forms of cancer which may offer effective oncological control and low morbidity. We performed a review of PubMed articles assessing the current applications of focused ultrasound in the treatment of genitourinary cancers, including prostate, kidney, bladder, penile, and testicular cancer. Current research indicates that high-intensity focused ultrasound (HIFU) focal therapy offers effective short-term oncologic control of localized prostate and kidney cancer with lower associated morbidity than radical surgery. In addition, studies in mice have demonstrated that focused ultrasound treatment increases the accuracy of chemotherapeutic drug delivery, the efficacy of drug uptake, and cytotoxic effects within targeted cancer cells. Ultrasound-based therapy shows promise for the treatment of genitourinary cancers. Further research should continue to investigate focused ultrasound as an alternative cancer treatment option or as a complement to increase the efficacy of conventional treatments such as chemotherapy and radiotherapy.

## 1. Introduction

### 1.1. Background

Cancer is an extremely prevalent disease, with an estimated 17 million new diagnoses and 9.5 million associated deaths worldwide in 2018 [1]. Genitourinary cancers represent a significant portion of cancer-related morbidity, with prostate cancer (PCa) as the fourth most common form of cancer globally [2]. Various forms of treatment for genitourinary cancer currently exist, such as hormonal therapy, surgery, chemotherapy, and radiotherapy [3]. While these treatments are clinically effective, they are often associated with significant morbidity for patients [4]. As such, a need exists for effective, less invasive treatment options for patients with genitourinary malignancies.

Focal therapy refers to the ablation of precisely targeted cancerous tissue, typically through producing extremely hot or cold temperatures [5]. Focal ablation has developed as a treatment option for managing various forms of localized cancer which is less invasive and has been associated with lower morbidity than surgical treatments [5]. Common focal therapy techniques include cryotherapy, focal laser ablation, radiation therapy, microwave therapy, and high-intensity focused ultrasound (HIFU) [5]. HIFU is a form of focal therapy which utilizes ultra-high-frequency soundwaves to heat and kill precisely targeted cancerous tissue while sparing the surrounding, healthy tissue [6]. While used most prominently in the treatment of localized Pca [6], ultrasound-based treatments of cancer, including HIFU therapy, have been explored and show promise for the treatment of other genitourinary malignancies as well.

### 1.2. Methods

The aim of this literature review is to provide an overview of the use of focused ultrasound therapy in the treatment of genitourinary cancers. The PubMed data base was searched for English language articles using queries with prostate or kidney cancer and focal therapy in the title from 2016 to 2022 and 2009 to 2022, respectively. In total, 161 results were returned, and articles were selected based on relevance and recency, as determined by the expertise of the authors. We sought to provide insight as to the current applications, efficacy, and clinical outcomes of focused ultrasound treatments, as well as the potential for future growth. Appropriate articles were summarized in the tables as determined by the authors’ discretion.

### 1.3. History of Ultrasound: Imaging Modality

The exploration of medically relevant uses of ultrasound began in the early 20th century, with unsuccessful attempts to examine brain tissue for the presence of tumors based on tissue density [7]. In 1950, Dr. John Wild reported on discrepancies in density of cancerous and healthy tissues [8] as well as the diagnostic implications of these findings [9]. Shortly after, ultrasound utility gained wide recognition among the medical community when Donald et al. (1958) [10] reported on the diagnosis of a benign cystic mass via ultrasound which was previously diagnosed as advanced gastric cancer through conventional tests. These advancements led to a booming interest in the medical applications of ultrasound throughout the 1960s, expanding its usage and catalyzing its development [11]. Today, ultrasound remains a prominent imaging modality with a wide variety of applications, including monitoring pregnancy development and assessing various tissues for pathology, including cancers.

### 1.4. History of Ultrasound: Treatment Modality

Early exploration of the ablative effects of focused ultrasound began in 1944, with Lynn and colleagues demonstrating the destruction of liver tissue and the induction cerebral structural and behavioral changes in live mice [12]. However, it was not until 1999 that Gelet et al. reported the first clinical investigation of ultrasound through high-intensity focused ultrasound (HIFU) focal therapy for the treatment of localized PCa [13]. Since then, the use of HIFU has expanded for treatment of a wide variety of medical conditions [14]. These include the destruction of targeted tissues [15], increasing membrane permeability for enhanced drug uptake, cytotoxic effects, and tumor death in cancerous tissues [16,17,18]. The inherent acoustic attributes of this modality have been utilized to direct drug-filled microbubbles towards desired tissues for more precise drug delivery and reduction of systemic toxicity [19]. Other common applications include the destruction of kidney stones [20] and temporary disruption of the blood–brain barrier for drug delivery [21].

## 2. Prostate Cancer

### 2.1. HIFU Focal Therapy

#### 2.1.1. Patient Selection

One of the most prominent uses of ultrasound treatment is HIFU focal therapy for ablation of localized PCa, which can occur through ablation of the entire prostate, ablation of half of the prostate (hemi-ablation), or the targeting of specific cancerous lesions [22]. When considering focal therapy for PCa, proper patient selection is crucial for maximizing the probability of successful outcomes. Expert consensus opinion gathered by Tay et al. (2017) states that patients with low risk and localized cancer with tumors smaller than 1.5 milliliters or below 20% of total prostate volume are most appropriate for focal therapy [23]. Rosette et al. (2010) also established a consensus panel which includes recommendations for the selection of low- to moderate-risk patients with stage cT2A disease or less [24], meaning the tumor involves less than one half of one lateral lobe of the prostate [25]. Caution was advised when considering focal therapy for the ablation of anterior or apical tumors, which could pose heightened risk of unsuccessful ablation [24]. Currently, the American Urological Association (AUA) states that focal therapy, including HIFU, should be considered an experimental modality only for the treatment of intermediate-high-risk localized PCa [26].

#### 2.1.2. Oncological Effectiveness and Follow-Up Period

A phase 2 trial conducted by Liu et al. (2021) [27] examined HIFU therapy among 44 men with intermediate-risk PCa and found that 93% of patients were disease-free on biopsy at 5 months following treatment [27]. In addition, no significant decline in erectile function was noted among the treated population [27]. A separate phase 2 trial conducted by Ganzer et al. (2018) [28] which assessed HIFU prostate hemi-ablation in 49 men reported that 26.5% of patients were biopsy-positive for PCa at 12 months, but only 8.2% were positive for clinically significant PCa [28] (Table 1). The study also noted that potency was preserved in 21 of 30 (70%) participants, and no change occurred in continence or quality of life for patients [28].

A pooled data study by Albisinni et al. (2018) [38], which assessed 366 cases of HIFU therapy for the treatment of unilateral PCa collected from seven different studies, demonstrated a negative biopsy rate of 87% one year following treatment and a salvage treatment free survival rate of 92% [38]. In addition, potency and continence were preserved in 74% and 96% of patients, respectively [38]. Similar research from Claros et al. (2019) [29] analyzed 59 patients treated with HIFU focal therapy with a median follow up period of 18 months. Failure-free survival (FFS) rates of 83% and 74% were reported at 2 and 4 years, respectively. The authors also report 3-month potency and continence rates of 75% and 93.4%, respectively, with a 23% overall complication rate [29]. Additional research assessing whole-gland ablation in 1002 patients conducted by Crouzet et al. (2014) [30] reports 8-year biochemical-free survival (BFS) of 76%, 63%, and 57%, for low-, intermediate-, and high-risk patients, respectively [30]. The study also reports a high 10-year metastasis-free survival (MFS) rate of 94% [30]. Guillaumier and colleagues (2018) [31] also report high success rates among 625 patients treated with HIFU of specifically targeted areas of the prostate. According to the study, patients displayed FFS rates of 99% at 1 year, 92% at 3 years, and 88% at 5 years [31]. In addition, the MFS, cancer specific survival (CSS), and overall survival (OS) rates at 5 years were 98%, 100%, and 99%, respectively [31]. Brundl et al. (2021) [39] offer the longest follow-up data, with 560 patients assessed 21 years following whole-gland HIFU ablation. At a median follow up of 15.1 years, the CSS rates for low-, intermediate-, and high-risk patients were 95%, 89%, and 65%, respectively [39]. The MFS and BFS rates were also favorable, with MFS rates 91%, 85%, and 58% and BFS rates of 82%, 73%, and 47% for the low-, intermediate-, and high-risk groups, respectively [39]. When comparing whole- and partial-gland ablation, a study conducted by Byun et al. (2022) found similar rates of cancer-free postoperative biopsy (70.4% vs. 72.9%) and erectile dysfunction-free survival (*p* = 0.317) but significantly shorter incontinence-free survival among the partial gland ablation group (*p* = 0.047) [40]. Additionally, patients who received partial-gland ablation demonstrated significantly lower rates of postoperative complications and additional endoscopic surgery for bladder obstruction compared to whole-gland patients (37.5% vs. 66.7%, *p* = 0.023 and 15.8% vs. 35.2%, *p* = 0.005, respectively) [40].

### 2.2. MRI-Guided HIFU Therapy

MRI is being explored as a means to guide HIFU therapy, with the potential benefits of enhanced visualization of soft tissues, near real-time heat mapping, and enabling a targeted approach for focal ablation [41]. Initial studies indicate that MRI-guided HIFU offers a safe and effective technique for ablation of prostate tumors. Nair et al. (2021) assessed MRI-guided transurethral ultrasound ablation of prostate cancer among 30 patients and report an estimated 3 year biochemical-free survival rate of 74% with low overall morbidity and no significant difference in urinary incomitance, erectile function, or quality of life at 1 or 3 years following treatment compared to baseline [42]. Similarly, a phase 1 clinical trial involving 20 patients undergoing MRI-guided transurethral prostate ablation by Chin and colleagues (2016) demonstrated no intraoperative complications, a pad-free continence rate of 100% at 12 months, and an 87% decrease in median prostate specific antigen (PSA) from 5.8 ng/mL at baseline to 9.8 ng/mL at 1 month and remining stable to 12 months [43]. A different phase 1 clinical trial assessing the safety of MRI-guided transrectal HIFU across 8 patients reports 0 major adverse events and no change in the urinary function of 7 out of 8 patients between baseline and 6 months post-treatment [44]. Additionally, all 8 patients demonstrated negative MRI findings, 6 out of 10 treated regions returned negative biopsy results, and mean PSA declined from 5.06 ng/mL at baseline to 3.41 ng/mL 6 months afterwards [44]. The safety of this procedure is further illustrated by Burtnyk et al. (2015), who followed 8 patients for 28 days after transurethral MRI-guided prostate ablation and report no adverse events and no fecal or urinary retention or incontinence detected through routine monitoring [45].

### 2.3. HIFU Complications

Literature has shown that HIFU, as well as other forms of focal therapy, are generally well-tolerated by patients with low operative morbidity [46]. Still, numerous complications could arise from treatment with HIFU therapy, which include acute urinary retention, erectile dysfunction, urinary incontinence, urethral sloughing, urinary tract infection, urethral stricture, hematuria, and recto-urethral fistula [46,47]. Reported complications from HIFU focal therapy are reported in Table 1.

Schmid et al. (2019) [35] assessed the postoperative complications among 98 men undergoing HIFU focal therapy for low- and intermediate-risk PCa. The authors report a 35.7% complication rate within the first 30 days following treatment [35]. In addition, 26.5% of patients experienced urinary retention (UR), while 15.3% experienced urinary tract infections (UTI), and 6.1% experienced both [35]. Between 30 and 90 days the complication was very low, at 2%, with one patient experiencing UR and one patient developing a UTI [35]. Feijoo et al. (2016) [36] also describes low complication rates among 67 men treated with HIFU focal therapy hemi-ablation. According to the study, patients experienced a 14% postoperative complication rate, with 4 UTIs and 6 cases of UR [36]. In addition, continence was preserved in all patients, and 11 of 21 patients maintained potency at 3 months following the procedure [36]. Additional research from Abreu and colleagues (2020) [32] analyzed 100 patients who underwent hemi-gland HIFU therapy and reported minor complications in 13% of patients and no major complications [32]. The complications reported include UR, UTI, and neuropraxia among 7%, 5%, and 1% of patients, respectively [32].

Van Velthoven et al. (2016) [33] similarly evaluated perioperative complications in 50 patients who received HIFU focal therapy for unilateral, low-, or intermediate-risk PCa. The authors report low overall morbidity, with patients experiencing acute urinary retention (8%), UTI (6%), lower urinary tract symptoms (18%), urethral stricture (4%), transient incontinence (14%), persistent incontinence (6%), and de novo erectile dysfunction in 20% of previously sexually active patients [33].

In a study comparing cryoablation and HIFU in 335 focal therapy patients, Bakavicus et al. (2019) [34] report an overall complication rate of 38% and 21% among HIFU and cryoablation patients, respectively [34]. Of these complications, 95% and 91% were considered minor among the HIFU and cryoablation treatment groups, respectively [34]. Complications among the 210 patients who received HIFU therapy include dysuria (3.8%), urethral sloughing/visible hematuria (8.1%), acute urinary retention (17%), urethral stricture (1.9%), hematospermia (1.4%), UTI (8.1%), acute infective epididymitis (4.8%), and pain, hypotension, and hydronephrosis/renal colic (0.5% each) [34].

### 2.4. Comparison of HIFU to Surgery

A substantial body of literature comparing HIFU focal therapy to conventional treatments, such as surgery and radiotherapy, has shown similar oncological effectiveness and reduced morbidity for patients.

Van son et al. (2021) [48] assessed 530 patients who underwent focal therapy for PCa, including HIFU, and compared the oncological outcomes with 830 patients who underwent radical therapy. The authors report no statistically significant difference in 6-year FFS between patients treated with focal therapy (72.8%) and those treated with radical therapy (80.3%) (*p* = 0.1) [48]. Shah et al. (2021) [49] also assessed outcomes of focal therapy patients, including both HIFU and cryotherapy, compared to radical prostatectomy (RP) for the treatment of non-metastatic prostate cancer. Results of this analysis showed no significant difference in FFS over the 8-year follow-up period between the groups, with 3-, 5-, and 8-year FFS rates of 86%, 82%, and 79% and 91%, 86%, and 83% for RP and focal therapy, respectively (*p* = 0.12) [49]. Additional research from Albisinni and colleagues (2017) [50] matched and compared 55 recipients of HIFU hemi-ablation and robotic RP for unilateral PCa. With a median follow up of 36 months, results showed no significant difference in the need for salvage treatment within the two groups (*p* = 0.76) [50]. In addition, HIFU patients showed a lower risk of erectile dysfunction and faster recovery of continence following treatment, with 82% displaying no signs of incontinence immediately after the procedure [50].

A multifaceted study conducted by Chiang and Liu (2016) [51] compared complications and oncological outcomes among patients who received robotic RP (97), high-dose-rate brachytherapy (HDRBT) (161), cryoablation (114), and whole-gland HIFU (120) for localized PCa. Among the treatments assessed, robotic RP demonstrated significantly higher rates of urethral stricture (29.9% vs. 6.2%, 3.3%, and 10.8% for HDRBT, cryotherapy, and HIFU, respectively, *p* = 0.000), secondary transurethral prostate resection or optical internal urethrotomy (28.9% vs. 11.2%, 8.8%, and 13.3%, *p* = 0.000), and urinary incontinence (11.3% vs. 0.6%, 1.6%, and 2.5%, *p* = 0.000). In addition, HIFU displayed the lowest rates of de novo erectile dysfunction at 1 year (65.6%) compared to all other treatments assessed. When considering oncological outcomes at similar mean follow-ups of approximately 33 months, HIFU displayed the lowest rate of biochemical recurrence (24.2%, *p* = 0.000), which was especially low among low- and intermediate-risk groups (6.7% and 8.5%, respectively). HIFU treatment also resulted in the highest average number of BFS months (27.66 months%, *p* = 0.000), the highest MFS rate (99.2% *p* = 0.001), and demonstrated a salvage-free treatment higher than RP (70.0% vs. 60.8%, respectively, *p* = 0.000). When considering quality of life outcomes, the authors reported no significant difference in the quality of life among the four treatment groups at 12 months following the operation (*p* = 0.097) or beyond.

Hatiboglu et al. (2017) [52] also assessed quality of life in 130 HIFU patients and report no significant change in the overall quality of life of patients prior to and after receiving HIFU focal therapy [52]. Similarly, Royce et al. (2020) [53] evaluated quality of life in 70 men following HIFU and report no significant change in quality of life from baseline and at 12 and 24 months following surgery [53]. Data comparing quality of life outcomes of patients who receive HIFU therapy to other treatment modalities are limited. However, one study conducted by de Cerquiera et al. (2015) reports that patients undergoing active surveillance demonstrated lower health perception scores and increased hopelessness compared to patients receiving focal cryoablation [54]. Assessing non-focal modalities, a systematic review comparing active surveillance, external beam radiation therapy, and surgery for quality of life impact found that active surveillance had the lowest impact on quality of life, while surgery presented elevated rates of urinary and sexual dysfunction and radiation therapy was associated with elevated bowel dysfunction [55]. Additionally, it is important to note that, as expressed in an integrative review by Dickey and Grayson (2019), a significant number of studies report generally low anxiety and improved erectile function among men undergoing active surveillance compared to other treatment modalities [56]. Therefore, further research is needed to clearly elucidate the comparative impact of treatment modality, including HIFU therapy, on the quality of life and mental health of prostate cancer survivors.

Despite these studies assessing outcomes for HIFU therapy, a number of studies still indicate potentially lower oncologic control compared to radical treatments. A large study conducted by Garcia-Barreras et al. (2017) [57] assessed 1458 patients who either underwent robotic-assisted RP or partial-gland ablation through either HIFU or cryoablation for the treatment of low- and intermediate-risk PCa. Overall, partial-gland ablation showed a higher probability of salvage treatment (*p* < 0.001), with lower salvage treatment-free survival at 20 months (86.2% vs. 97%) and 46 months (77% vs. 95.8%) compared to RP [57]. The results also indicate greater continence recovery following RP but lower potency rates at 3, 6, and 12 months post-operation [57]. Rosenhammer et al. (2019) [58] similarly compared whole-gland HIFU ablation with RP and report greater OS (*p* < 0.0001), CSS (*p* = 0.0023), BFS (*p* = 0.0236), and salvage treatment-free survival (*p* = 0.0132) among the patients who received RP (91%, 98%, 80%, 80%, and 76%, 94%, 70%, and 71% after RP and HIFU, respectively) [58]. Importantly, the authors report significantly lower CSS following HIFU among higher and intermediate-risk patients (*p* = 0.010 and *p* = 0.048, respectively) but no significant difference among those with low-risk PCa (*p* = 0.4890) [58].

Overall, the literature suggests that HIFU focal therapy offers effective oncological control of low- and intermediate-risk prostate cancer and reduced morbidity when compared with radical alternative treatments.

### 2.5. Salvage Therapy Following HIFU

When cancer recurrence occurs following initial HIFU focal therapy, salvage treatment may be necessary. Current research indicates that salvage surgery following HIFU is safe to perform but may be correlated with suboptimal oncological outcomes.

Herrera-Caceres et al. (2020) [59] assessed 34 men who underwent salvage RP following focal treatment of PCa, including 19 HIFU patients. Complications were minor, with no rectal or urethral injuries, and 11.8% of patients developed bladder neck stricture. However, 38% of patients still had positive margins, and 10.6% developed biochemical recurrence at a mean follow-up of 4.3 years [59]. In a similar study, Thompson and colleagues (2020) [60] assessed 45 men who underwent salvage robotic RP following HIFU therapy for adverse effects and oncological effectiveness. The complication rate was low, with Clavien-Dindo grade 1, 2, and 3 complications in 8.9%, 6.7%, and 2.2% of patients, respectively, but oncological effects were suboptimal, with only 66.7% of patients free from radiotherapy or androgen deprivation therapy at 12 months post-procedure [60]. Additional research comparing primary robotic-assisted RP to robotic-assisted RP for salvage treatment following focal therapy found no significant difference in the complication rate (*p* > 0.05) but a lower recovery of erectile function (*p* = 0.008) and a substantially lower rate of BFS among the salvage group (56.3% BFS among salvage vs. 92.4% among primary, *p* = 0.001) [61].

These studies indicate that salvage therapy following focal therapy is safe to perform, but the oncological outcomes are suboptimal compared to primary therapy of the same modality—an important consideration when assessing patient selection for primary focal therapy.

### 2.6. HIFU for Salvage Therapy

In addition to its role as a primary treatment modality for localized PCa, HIFU focal therapy has been investigated as a salvage therapy option for recurrence following unsuccessfully treated cancer treated by prior focal therapy or other modalities.

A literature review conducted by Maestroni et al. (2021) [62] assessed 1241 patients who underwent HIFU salvage therapy in radio-recurrent PCa. The authors report a 51.6% recurrence rate, regardless of the follow-up period, and an OS rate at 5 years of 85.2% [62]. Kanthabalan et al. (2017) [63] also assessed 150 men who underwent focal salvage HIFU utilizing a composite outcome based on biochemical failure, positive imaging or biopsy, the requirement of additional systemic therapy, metastases, or cancer-specific mortality. The analysis demonstrated a 3-year composite-free survival rate of 100%, 49%, and 24% for low-, intermediate-, and high-risk pre-salvage PCa, respectively [63]. Associated complications were low, with 11.3% of patients developing a UTI, 8% developing bladder neck stricture, and 2% developing a rectourethral fistula [63].

Research assessing salvage HIFU therapy following brachytherapy with local recurrence found 6-year FFS rates, progression-free survival, CSS, and MFS rates of 41%, 45%, 98%, and 80%, respectively [64]. Other research from Crouzet et al. (2017) [65] assessing 418 patients who underwent salvage HIFU after failed external beam radiation therapy and found 7-year OS, CSS, and MFS rates of 72%, 82%, and 81%, respectively [65]. In addition, BFS for low-, intermediate-, and high-risk PCa was reported at 58%, 51%, and 36%, respectively [65].

A comparison of HIFU and RP for salvage therapy of radio-recurrent prostate cancer conducted by Devos et al. (2019) [66] found no significant difference in the 5-year OS (*p* = 0.24), CSS (*p* = 0.36), and MFS (*p* = 0.55) rates between the two modalities [66]. However, HIFU patients experienced significantly lower rates of 12-month pad dependence (22.2% vs. 56.0%, *p* = 0.01) due to incontinence, and grade 3 complications (25.9% vs. 48.0%, *p* = 0.027) compared to RP patients [66].

### 2.7. Following Patients after HIFU Therapy

Currently, no standardized method for following and evaluating patients who receive HIFU focal therapy for PCa exists. However, experts recommend several strategies to monitor and assess patients to ensure recurrence and potential disease progression are promptly detected and addressed.

Muller et al. (2015) [67] performed a literature review and conducted a consensus study among 76 experts, including primarily urologists and radiologists, on recommendations for monitoring patients after focal therapy. The experts recommended assessments of erectile function, quality of life, and urinary symptoms on a regular basis, as well as utilizing multiparametric MRI-guided biopsies with systematic targeted biopsies at 1 year and upon the detection of suspicious imaging. They also recommended PSA testing every 3 months during the first year and every 6 months each year after [67]. Similarly, a systemic review performed by Tay and colleagues (2019) [68] assessed recommendations of surveillance following focal therapy for PCa. The recommendations included multiparametric MRI imaging at 6 months, between 12 and 24 months, and 5 years following treatment [58]. In addition, the authors recommend targeted biopsy of the treated zone and suspicions lesions at 3 to 6 months and systematic biopsy at 12 to 24 months and at 5 years post-treatment [68]. The authors also mention potential utility in monitoring PSA levels following treatment, but the lack of data on typical patterns and outcomes of PSA changes after focal therapy makes its current role unclear. Current research from Stabile et al. (2020) [69] determined that the percent reduction in PSA is a significant predictor of the need for additional and radical treatment. The authors report that patients with a 90% or greater reduction in PSA had a less than 20% probability of needing additional treatments within 5 years, whereas patients with a 20% reduction or less had a greater than 70% probability of needing additional treatment within 5 years [69]. Research investigating other modalities suggest that contrast-enhanced ultrasound [70,71] and multiparametric MRI are effective tools in monitoring patient progression and detecting the recurrence of PCa as well [72].

### 2.8. Limitations of HIFU Focal Therapy

There are several important drawbacks to consider when evaluating patients for treatment with HIFU focal therapy. Despite promising results in low- and intermediate-risk patient groups, the oncological effectiveness is substantially inferior among high-risk cancer groups. Research from Callea et al. (2010) [73] assessed oncologic outcomes of 171 patients undergoing HIFU and found that, at a mean follow up of 67.9 months, the rate of BFS was 84.2% among low- and intermediate-risk patients but only 43.1% among high risk patients [73]. Additionally, 93.4% of low- and intermediate-risk patients showed no residual tumor on a 6-month biopsy, while only 63.1% of high-risk individuals were tumor-free [73].

Current literature also suggests that ablation of anterior tumors may be significantly less effective than posterior tumors. Dellabela et al. (2021) [37] evaluated 189 patients treated with HIFU focal therapy and found that the risk of failure was three times higher for cancers in the anterior stroma of the prostate compared to other locations (odds ratio (OR) 3.36, 95% confidence interval (CI) 1.18–9.53) [37]. Similarly, Huber et al. (2021) [74] assessed 267 patients, 45 with anterior focal and 222 with posterior focal HIFU, and found that 37.8% of anteriorly located tumors required further treatment compared to 20.3% of posteriorly located cancers [74].

Additionally, the risk of suboptimal oncologic outcomes in the case of salvage therapy following HIFU focal therapy may be higher when compared to primary radical prostatectomy. This needs to be considered when evaluating patients for HIFU as a first line treatment [60,61].

Given the lack of long-term comparative data on HIFU focal therapy, both the American Urological Association (AUA) and the European Association of Urology (EAU) currently recommend only offering HIFU focal therapy in the context of a clinical trial [75,76].

## 3. Kidney Cancer

### 3.1. Ablative Focal Therapy

Ablative therapy for the treatment of localized renal carcinoma is growing in popularity due to early indications of effective oncological control and low morbidity. In the context of renal cancer, focal therapy generally refers to complete tumor ablation through minimally invasive means such as HIFU or cryoablation while sparing healthy tissues of the kidney. Collectively, research on various techniques for the focal ablation of kidney cancer have shown positive outcomes, indicating the utility of focal therapy in the treatment of small renal masses.

### 3.2. HIFU in Renal Cancer: Animal Model Results

Numerous studies in animal models served as early indications for the efficacy of HIFU kidney ablation. Animal models previously assessed have included pigs [77,78] and rabbits [79,80] among others. Overall, the results from these studies indicate that HIFU therapy administered noninvasively can successfully ablate kidney tissues, including native or cancerous cells, with minimal toxicity to surrounding tissues.

### 3.3. HIFU in Renal Cancer: Human Results

Although HIFU therapy has not been investigated as extensively as other ablative therapies such as radio frequency ablation (RFA) and cryotherapy, proof of concept research has shown that laparoscopic HIFU can achieve successful tissue ablation with low associated morbidity [81]. An early study conducted by Vallancien et al. (1992) assessing five patients with renal cancer treated with transrectal HIFU found that, upon histological evaluation, all five patients showed lesions with high congestion, hyperemia, and alteration to microcapillaries, indicative of successful ablation [82]. An additional single center study which followed two patients who received HIFU ablation for renal cell carcinoma in transplanted kidneys reported no recurrent tumors at 73 and 81 months follow-up and minimal associated morbidity [83].

In addition to its potential for tissue ablation, research has indicated a potential immunotherapeutic benefit of HIFU therapy. A study conducted by Ritchie et al. (2010) assessed immune response following HIFU ablation of renal tumors and found that myeloid-derived suppressor cell population declined following the procedure [84]. This indicates that HIFU therapy may promote enhanced anti-tumor immune response through decreasing these T-cell suppressors and could potentially reduce the likelihood of metastatic disease [84].

Despite the promising results from noninvasive HIFU ablation trials in animal models and limited human applications, there are drawbacks to consider. Most prevalently, studies have indicated that extracorporeal HIFU for the treatment of renal masses can show varied reliability, due to variables such as respiratory movements, ribs, and perirenal structures and fat [85,86].

### 3.4. Focal Therapy Oncological Success Rate/Follow-Up Time

In a study assessing cancer-specific mortality following local focal ablation among 1860 patients with clinical stage T1A (cT1A) kidney cancer, meaning the tumor is confined to the kidney and does not exceed 4 cm on computerized tomography imaging [87], Larcher et al. (2016) [88] report a significantly lower rate of cancer specific mortality among focal therapy patients compared to observation alone (hazard ratio 0.47, 95% CI 0.25–0.89, *p* = 0.02) [89]. To compare the efficacy of RFA and cryoablation for the treatment of small renal masses, Kunkle and Uzzo (2008) [89] performed a literature review including over 1300 patients and concluded that both methods offer effective short term oncological control [89]. However, at a mean follow up of 18.7 months, cryoablation was associated with significantly lower rates of tumor progression (5.2% vs. 12.9%, *p* < 0.0001) and repeat ablation (8.5% vs. 1.3%, *p* < 0.001) compared to RFA [89] Goel and Kaouk (2008) [90] also compared cryotherapy and RFA [90], reporting similar 3-year CSS rates of 98% and 97%, respectively [90]. However, cryoablation displayed a lower local recurrence rate (4.6% vs. 11.7%), metastatic progression rate (1.2% vs. 2.3%), and retreatment rate (0.9% vs. 8.8%) compared to RFA [90]. Oncological and operative outcomes of focal therapy for renal masses are displayed in Table 2.

### 3.5. Focal Therapy Comparisons to Partial Nephrectomy

Currently, partial nephrectomy (PN) is considered the standard of care for treatment of small renal masses [98]. However, focal therapy could offer a minimally invasive alternative to surgery with similar oncological outcomes and reduced morbidity for patients.

Salagierski et al. (2018) [99] performed a literature review comparing the efficacy of focal ablative therapy and partial nephrectomy for the treatment of small renal masses. The authors concluded that focal therapy offers comparable oncologic control for renal tumors smaller than 3 cm while maintaining excellent cost effectiveness and fewer postoperative complications compared to PN [99]. Research from Choueiri and others (2011) [100] support these findings in a study which investigated 15,145 patients who underwent either thermal ablation (578), PN (4402), or radical nephrectomy (RN) (10,165) for renal cell carcinoma. Analyses showed no significant difference in adjusted 2-year CSS (RN vs. TA *p* = 0.7, PN vs. TA *p* = 0.2) or 2-year OS (RN vs. TA *p* = 0.73, PN vs. TA *p* = 0.32) between the groups (98.0%, 99.3%, and 98.0% and 92.5%, 97.6%, and 94.6% for thermal ablation, PN, and RN, respectively) [100].

Similarly, Andrews et al. (2019) [101] assessed 1798 Mayo Clinic patients treated for renal masses between 2000 and 2011, comparing PN, RFA, and cryoablation for oncological outcomes. Among 1422 cT1A patients, no significant difference in 5-year CSS rate was found among those treated with PN (99.3%) compared to those treated with RFA (95.6%, *p* = 0.5) or treated with cryoablation (100%, *p* = 0.4) [101]. Furthermore, the 5-year local recurrence-free survival rate among 376 CT1B patients was not significantly different among patients treated with PN (93%) compared to cryoablation (95%, *p* = 0.8), nor was the 5-year metastasis-free survival rate (94% vs. 90% for PN and cryoablation, respectively, *p* > 0.9) [101].

In another study investigating 1424 patients with cT1A renal cell carcinoma treated with either PN, RFA, or cryotherapy, Thompson et al. (2014) [102] found no significant difference in the 3-year recurrence-free survival rate among the treatments (98% for all three groups, *p* = 0.49) [102]. However, the MFS rate was significantly higher among the cryoablation (*p* = 0.021) and PN (*p* = 0.005) groups compared to RFA (100%, 99%, and 93% for cryoablation, PN, and RFA, respectively). Among 379 CT1B patients, no significant difference was reported in recurrence-free survival (*p* = 0.81) and MFS (0.45) between PN and cryoablation [102]. Other research investigating RFA and PN among patients with CT1B renal cell carcinoma reports non-significantly different 5-year CSS (*p* = 0.493) and disease-free survival (*p* = 0.364) rates among the treatment groups. (92.6% and 81.0% vs. 96.6% and 89.7% for RFA and PN, respectively) [103].

PN has been associated with complication rates as high as 33% [104] and morbidities including infection, hemorrhagic bleeding necessitating blood transfusions, and urinary leakage [105]. Research has shown that ablation therapy can reduce the risk of complications in high-risk patients [104].

Separate literature reviews performed by Hu et al. (2019) [106] and Rivero et al. (2018) [107] compared complication rates between patients undergoing thermal ablation and PN for kidney cancer. Both studies reported fewer adverse effects among patients who received thermal ablation than PN, with complication rates of 12.53% and 17.00% for the Hu study [106] and 13% and 17.6% for the Rivero study [107] for ablation and PN, respectively.

Larcher et al. (2017) [92] investigated perioperative complications in 2471 cT1A kidney cancer patients who received partial nephrectomy or local ablation treatment. The authors report an overall complication rate of 21% for local ablation compared to 40% following partial nephrectomy (*p* < 0.001), with reduced length of stay (*p* < 0.05) and healthcare expenditures (*p* < 0.05) for local ablation and no significant difference in readmission rate (*p* = 0.1) or 30-day mortality rates (*p* = 0.2) [92]. In a similar study, Pantelidou et al. (2016) [108] assessed 126 patients, 63 of whom underwent RFA and 63 of whom underwent robotic-assisted PN, and found that the minor complication rate was similar between robotic PN and RFA (6.35% and 15.87%, respectively, *p* = 0.15), but postoperative renal function decline was significantly lower among patients treated with RFA (*p* < 0.0001) [108]. An investigation from Talenfeld et al. (2018) [93] followed 4310 patients for a median of 52 months, comparing percutaneous ablation with PN and RN for renal cell carcinoma, and found that percutaneous ablation and PN were associated with lower renal insufficiency than RN (11% and 9% vs. 18%, respectively, *p* = 0.005). Additionally, ablation treatment displayed a lower 30-day non-urological related complication rate and acute renal failure than the other treatment modalities (6% vs. 29% and 30% and <3% vs. 7% and 11% for ablation, PN, and RN, respectively) [93]. An additional comparison of patients treated with laparoscopic PN and RFA from Ruiz and colleagues (2019) [96] found a substantially higher complication rate following laparoscopic PN than RFA (43.9% vs. 10.7%, respectively, *p* < 0.001), including a higher prevalence of infection- and bleeding-related complications [96]. These findings are supported by Guan et al. (2012) [97], who compared PN and microwave ablation patients and found that patients who underwent microwave ablation experienced reduced blood loss (*p* = 0.002), lower decline in renal function (*p* = 0.0092), and a lower complication rate (*p* = 0.0187) while maintaining no significant difference in recurrence-free survival (*p* = 0.5414) [97].

In addition to the reduced complication rate, research suggests that the financial burden from focal therapy is substantially lower compared to PN. Chehab et al. (2015) [109] compared the costs of cryoablation, open, and robotic PN and found a significantly lower cost for cryoablation ($6067 vs. $11,392 and $11,830, respectively, *p* < 0.0001) [109]. Focal therapy was also associated with lower cost of procedure room (*p* < 0.0001), anesthesia (*p* < 0.0001), patient room and board (*p* < 0.0001), laboratory fees (*p* < 0.0001), and shorter hospitalization time (*p* < 0.0001) compared to open and robotic PN [109].

### 3.6. Salvage Focal Therapy

Aside from use in the primary treatment of kidney cancer, focal ablation may serve a useful purpose as a minimally invasive option for salvage therapy following renal tumor recurrence. Early research indicates effective oncological control and minimal morbidity following ablative therapy for recurrent renal carcinoma. One such study from Yang et al. (2013) [110] assessed probe ablation as salvage therapy following partial nephrectomy in von Hippel-Lindau patients. Among the 14 patients assessed, the authors report no intraoperative or perioperative complications and minimal impact on renal function following the procedure [109]. At a mean follow up of 37.6 months, only three patients had undergone re-ablation for suspicious lesions, and the OS and CSS rates were 92% and 100%, respectively [110]. A small study conducted by Morgan et al. (2013) [111] assessing five patients treated with cryoablation following cancer recurrence after PN reached similar conclusions. At a mean follow up of 32 months, only one patient had developed additional recurrence, which was successfully treated with an additional round of cryoablation [111]. Additionally, the associated complication rate was low, with only two cases of successfully managed hematuria [110]. In an assessment of 20 patients undergoing repeat cryoablation due to recurrence following primary cryoablation, Okhunov et al. (2016) [112] reports no complications as well as local recurrence-free survival, MFS, and CSS rates of 85%, 100%, and 100%, respectively (30-month median follow-up) [112]. Overall, these studies serve as early indications that focal therapy can provide safe and effective salvage therapy for recurrent prostate tumors.

### 3.7. Focal Therapy Contraindications/Limitations

Several important limitations exist in the use of focal therapy in kidney cancer which must be considered when evaluating patient eligibility. Substantial literature currently exists indicating that focal ablation of large tumors yields poor oncological effectiveness when compared to small tumors. Research from Kim et al. (2013) [94] found tumor size greater than 3 cm to be a significant predictor of cryoablation failure, with a hazard ratio of 3.21 (95% CI 1.09–9.44, *p* = 0.03) [94]. Similarly, Johnson and colleagues (2019) [113] report a decline in 6-year recurrence-free survival and MFS from 97% and 100%, respectively, among tumors smaller than 3 cm, to 68% and 86% among tumors larger than 3 cm [113]. Additional findings from Best et al. (2012) [114] indicate a recurrence rate of approximately 20% for tumors larger than 3 cm and a decline in disease-free survival rate from 95% to 79% when comparing tumors smaller and larger than 3 cm, respectively [114].

In addition to size as a potential contraindication, limited literature suggests that cancer cell type may also influence the success rate of focal therapy for renal tumors. Lay et al. (2015) [115] report a significantly lower 5-year disease-free survival rate following RFA in clear cell and papillary tumors at 89.7% and 100%, respectively [115]. Tumor location is also important to consider, as masses located around certain delicate structures, such as the hilum and renal pelvis, are also contraindications for focal therapy [116].

### 3.8. Focal Therapy Guidelines

Currently, the AUA recognizes thermal ablation as a potentially viable alternative to surgical intervention among appropriately selected patients. The AUA recommends that physicians consider thermal ablation for the treatment of cT1A tumors smaller than 3 cm and recommends percutaneous over surgical approach when possible [117]. Currently, the AUA endorses RFA and cryoablation as ablative treatment, which clinicians may recommend, but HIFU is still considered an investigational modality [117]. Recommendations also include counseling patients about the potentially higher risk of cancer recurrence associated with ablative treatment compared to standard surgical treatments and recommending conducting a renal mass biopsy prior to initiating focal therapy [117]. EAU guidelines currently recommend offering thermal ablation to patients considered too frail or with comorbidities that serve as contraindications to surgery [118]. Additionally, it is not recommended to routinely offer thermal ablation for the treatment of tumors larger than 3 cm or to offer cryoablation specifically for the treatment of tumors larger than 4 cm [118]. National Comprehensive Cancer Network guidelines currently only recommend consideration of ablative therapy in patients with tumors 3 cm or smaller and in which partial nephrectomy is contraindicated [119].

## 4. Bladder Cancer

### 4.1. Standard Treatments

Current standard of care for the treatment of non-muscular invading bladder cancer includes transurethral tumor resection, intravesical Bacillus Calmette–Guerin, or intravesical chemotherapy [120]. For muscle-invasive or advanced bladder cancer, radical cystectomy, often preceded by neoadjuvant chemotherapy, is considered the standard of care [120,121].

### 4.2. HIFU Ablation

Laser ablation of bladder cancerous lesions has been shown to be effective with limited morbidity [122], but the application of HIFU therapy in bladder cancer is limited. An experimental study conducted by de Castro et al. (2016) assessed the efficacy of robotic-assisted laparoscopic HIFU ablation of simulated tumors made of striated muscle in cadaver bladders and found this technique was effective for bladder wall tumors [123], indicating the potential for using HIFU therapy in treating bladder tumors. However, this is not standard of care and is still considered an experimental treatment modality, requiring significantly more data before being utilized on a regular basis.

### 4.3. Microbubbles for the Delivery of Radiation and Chemotherapy

In addition to HIFU focal ablation, the unique biological and acoustic effects of focused US have been investigated for the enhancement of anticancer treatment efficacy. One such effect is the guidance of chemotherapeutic-filled microbubbles for enhanced drug delivery to cancerous tissues. Microbubbles are microscopic bubbles, typically coated with phospholipid shell, which can be used either as a contrast agent for contrast-enhanced ultrasound imaging [124] or as vessels for the directed delivery of chemotherapy [19]. Through ultrasound, these microbubbles can be directed towards cancerous tissue, increasing the accuracy of drug delivery and decreasing the systemic toxicity of the drugs [19]. Focused ultrasound also induces sonoporation in tissues, creating temporary pores in cell membranes through the oscillation of microbubbles at low ultrasound frequency (stable cavitation) or the rapid explosion and reformation of microbubbles (inertial cavitation) [16]. Sonoporation has been experimentally shown to increase the efficacy of drug uptake [16], potentially increasing the efficacy of chemotherapeutics. Research in mice grafted with human bladder cancer xenograft showed reduced tumor vasculature and enhanced cell death when treated with HIFU microbubble treatment, which was further enhanced when combined with radiotherapy [18]. Other research in mice with urethral carcinoma found that ultrasound beams were effective in guiding oxygen nanobubbles to tumors, enhancing the delivery of mitomycin-C and reducing tumor progression while utilizing lower drug concentrations [125]. Similarly, a study analyzing non-muscular invading bladder cancer in a 3D cancer mimicking culture found that ultrasound-induced microbubble cavitation enhanced cisplatin delivery, resulting in heightened drug concentration and cytotoxic effects within the targeted tumor cells [17]. However, it is important to note that despite promising experiential results, no clinical experience enhancing drug delivery has been described thus far.

## 5. Penile Cancer

Currently, ultrasound use in the diagnosis and treatment of penile cancer is very limited [126]. While laser ablation has been used effectively in the local treatment of selected squamous cell carcinomas [127], traditional treatments such as organ preserving surgery and localized radiotherapy for early-stage cancer and lymphadenectomy and chemotherapy for metastatic cancer are likely to remain the standard of care [128].

## 6. Testicular Cancer

Ultrasound imaging is the primary modality for the detection and assessment of testicular and extra testicular masses [129]. Research has shown that patients screened with ultrasound during routine follow-ups following testicular cancer treatment were often detected with smaller tumors and received more organ sparing surgery and organ preservation than those who were only screened for palpable tumors [130]. Additionally, ultrasound imaging has been shown to be effective for both preoperative imaging and intraoperative surgical guidance in the treatment of small and unpalpable lesions of the testes [131,132]. Very little research has demonstrated a utility for HIFU focal therapy in treating testicular cancer. Given the greater than 90% cure rate of testicular cancer through other more radical treatments, such as surgery and chemotherapy, it is unlikely that HIFU therapy will become a standard of care in testicular cancer.

## 7. Conclusions

### 7.1. Current Uses

Focused ultrasound has shown tremendous potential as a less invasive means for treating various forms of localized genitourinary cancers, especially prostate cancer. Specifically, the capacity of focused ultrasound to selectively target and ablate regions of cancerous tissue while sparing surrounding healthy tissue, as well as enhance the delivery of chemotherapeutic drugs through sonoporation and microbubble cavitation, make it a promising modality for standalone treatment in appropriate patients or for use in conjunction with other forms of therapy.

### 7.2. Future Directions

Ultrasound is a highly useful tool for diagnosis, biopsy, and focal treatment of prostate cancers. While ultrasound-based treatment is not currently as prevalent for the treatment of other forms of genitourinary cancers, it has shown tremendous potential and its use is likely to expand in kidney cancer as well, as more data become available and techniques become refined. Additionally, the role of HIFU for induced immunotherapy may expand, given its potential to reduce the likelihood of metastatic disease progression. The expansion of ultrasound-based treatments will likely provide a non-surgical alterative for patients with localized cancer which provides effective oncological control and lower associated morbidity.

## Figures and Tables

**Table 1:** Oncological and operative outcomes of HIFU focal therapy for the treatment of prostate cancers.

Study	Patients, *n*	Median Age (Years)	Mean Follow-up (Months)	Mean Pre-HIFU PSA (ng/mL)	Mean Post-HIFU PSA (ng/mL)	Failure Free Survival Rate, %	Overall Complication Rate, %	Urinary Retention, *n* (%)	Urinary Tract Infection, *n* (%)	Fistula, *n* (%)	Potency, %	Continence, %
Ganzer et al. (2018) [28]	51	63.4	17.4	6.2	2.9	73.5 (91.8 for csPCa)	41.2%	1(1.96)	9 (17.64)	-	70%	94.1%
Claros et al. (2019) [29]	59	66.7	18	7.6	2.67	72.9	23%	6 (10.1)	-	-	75%	93.4%
Crouzet et al. (2014) [30]	1002	71	76.8	7.7	0.14	78.8	66.57%	76 (7.6)	39 (3.9)	4 (0.4)	42.3%	76.3%
Guillaumier et al. (2018) [31]	625	65	56	7.2	-	88	20.4%	-	53 (8.5)	2 (0.4)	-	98%
Abreu et al. (2020) [32]	100	65	20	5.9	1.3	92	13%	7 (7)	5 (5)	0 (0)	-	100%
van Velthoven et al. (2016) [33]	50	73	39.5	6.6	1.6	58	36%	4 (8)	3 (6)	-	80%	94%
Bakavicus et al. (2019) [34]	210	68	11	7.4	-	-	38%	36 (17)	17 (8.1)	0 (0)	-	-
Schmid et al. (2019) [35]	98	66	3	6.5	-	-	35.7%	27 (27.5)	16 (16.3)	-	-	-
Feijoo et al. (2016) [36]	67	70.2	12	6.1	2.6	83.6	14%	6 (9.0)	4 (2.8)	-	52.4%	100%
Dellabela et al. (2021) [37]	189	70	29	5.8	2.2	88.1	33.3%	15 (7.9%)	-	1 (0.5%)	-	98.9%

PSA, prostate specific antigen; HIFU, high intensity focused ultrasound; ng/mL, nanograms per milliliter; csPCa, clinically significant prostate cancer.

**Table 2:** Oncological and operative outcomes of focal therapy for the treatment of renal masses.

Study	Number of Patients, *n*	Median Age, Years	Mean Follow-Up (Months)	Ablation Technique	Failure-Free Survival Rate, %	Metastasis-Free Survival Rate, %	Cancer-Specific Survival, %	Repeat Ablation Rate, %	Overall Complication Rate, %
Laguna et al. (2009) [91]	144	70.5	-	Laparoscopic cryoablation	-	-	-	-	15.5
Kunkle & Uzzo (2008) [89]	600	66.3	22.5	Cryoablation	94.8	99	-	1.3	-
Kunkle & Uzzo (2008) [89]	775	67.8	15.8	Radiofrequency ablation	87.1	97.5	-	8.5	-
Goel & Kaouk (2008) [90]	-	-	28.3	Cryoablation	95.4	98.8	98	0.9	10.0
Goel & Kaouk (2008) [90]	-	-	26.6	Radiofrequency ablation	88.3	97.7	97	8.8	7.0
Larcher et al. (2017) [92]	510	76	-	Local tumor ablation	-	-	-	-	21.0
Talenfeld et al. (2018) [93]	456	-	52	Percutaneous ablation	-	-	95	-	-
Kim et al. (2013) [94]	124	72.6	30.2	Percutaneous cryoablation	87	-	100	-	9.0
Atwell et al. (2015) [95]	46	73	60	Percutaneous cryoablation	-	97.2	94%	-	17.4
Ruiz et al. (2019) [96]	84	66	20.5	Radiofrequency ablation	80	-	-	-	10.7
Guan et al. (2012) [97]	48	45.5	32	Microwave ablation	95.8	-	100	-	12.5

## Abbreviations

PCa	Prostate Cancer
HIFU	High-Intensity Focused Ultrasound
FFS	Failure-Free Survival
BFS	Biochemical-Free Survival
MFS	Metastasis-Free Survival
CSS	Cancer-Specific Survival
OS	Overall Survival
UR	Urinary Retention
UTI	Urinary Tract Infection
RP	Radical Prostatectomy
HDRBT	High-Dose-Rate Brachytherapy
PSA	Prostate Specific Antigen
OR	Odds Ratio
CI	Confidence Interval
RFA	Radiofrequency Ablation
PN	Partial Nephrectomy
RN	Radical Nephrectomy
